# Parvovirus B19 Infection and Pregnancy: Review of the Current Knowledge

**DOI:** 10.3390/jpm14020139

**Published:** 2024-01-26

**Authors:** Fernanda Parciasepe Dittmer, Clara de Moura Guimarães, Alberto Borges Peixoto, Karina Felippe Monezi Pontes, Maria Paola Bonasoni, Gabriele Tonni, Edward Araujo Júnior

**Affiliations:** 1 Department of Obstetrics, Paulista School of Medicine, Federal University of São Paulo (EPM-UNIFESP), São Paulo 04023-062, SP, Brazil; fernanda.dittmer@unifesp.br (F.P.D.); cguimaraes@unifesp.br (C.d.M.G.); karina.pontes@unifesp.br (K.F.M.P.); araujojred@terra.com.br (E.A.J.); 2 Gynecology and Obstetrics Service, Mário Palmério University Hospital, University of Uberaba (UNIUBE), Uberaba 38050-501, MG, Brazil; alberto.peixoto@uftm.edu.br; 3 Department of Gynecology and Obstetrics, Federal University of Triângulo Mineiro (UFTM), Uberaba 38025-440, MG, Brazil; 4 Service of Gynecology and Obstetrics, Ipiranga Hospital, São Paulo 04262-000, SP, Brazil; 5 Department of Pathology, Santa Maria Nuova Hospital, Istituto di Ricovero e Cura a Carattere Scientifico (IRCCS), AUSL Reggio Emilia, 50122 Reggio Emilia, Italy; paolabonasoni@yahoo.it; 6 Department of Obstetrics and Neonatology, Istituto di Ricovero e Cura a Carattere Scientifico (IRCCS), AUSL Reggio Emilia, 42122 Reggio Emilia, Italy; 7 Discipline of Woman Health, Municipal University of São Caetano do Sul (USCS), São Caetano do Sul 09521-160, SP, Brazil

**Keywords:** parvovirus B19, intrauterine infection, anemia, Doppler, intrauterine transfusion

## Abstract

Parvovirus B19, a member of the *Parvoviridae* family, is a human pathogenic virus. It can be transmitted by respiratory secretions, hand-to-mouth contact, blood transfusion, or transplacental transmission. Most patients are asymptomatic or present with mild symptoms such as erythema infectiosum, especially in children. In rare cases, moderate-to-severe symptoms may occur, affecting blood cells and other systems, resulting in anemia, thrombocytopenia, and neutropenia. Non-immune pregnant women are at risk for fetal infection by parvovirus B19, with greater complications if transmission occurs in the first or second trimester. Infected fetuses may not show any abnormalities in most cases, but in more severe cases, there may be severe fetal anemia, hydrops, and even pregnancy loss. Maternal diagnosis of intrauterine parvovirus B19 infection includes IgG and IgM antibody testing. For fetal diagnosis, PCR is performed through amniocentesis. In addition to diagnosing the infection, it is important to monitor the peak of systolic velocity of the middle cerebral artery (PVS-MCA) Doppler to assess the presence of fetal anemia. There is no vaccine for parvovirus B19, and fetal management focuses on detecting moderate/severe anemia by fetal PVS-MCA Doppler, which, if diagnosed, should be treated with intrauterine transfusion by cordocentesis. Prevention focuses on reducing exposure in high-risk populations, particularly pregnant women.

## 1. Introduction

Parvovirus B19 is a virus that belongs to the family *Parvoviridae* and the genus Erythrovirus and is the only virus in this family known to be pathogenic in humans [1]. As the name of its genus suggests, it depends on blood cells (erythroid) to replicate [2].

The infection caused by parvovirus B19 (ICD10: O98.5) is transmitted through respiratory secretions, hand-to-mouth contact, blood transfusion, and transplacental transmission [3]. Because of the way it is transmitted, it is a common infection in childhood and is known as erythema infectiosum (EI) or “fifth disease”. Parvovirus B19 infection can be asymptomatic, but due to its tropism for blood cells, it may cause moderate-to-severe symptoms associated with anemia, depending on the immune and hematologic status of the host [4]. Parvovirus B19 can also affect other systems, eventually leading to thrombocytopenia and neutropenia [5].

Asymptomatic or mild infection is more common when parvovirus B19 infects immunocompetent adults. In pregnant women, fetuses in the first and second trimester are more susceptible to hydrops or demise, because erythrocyte progenitors develop primarily during these gestational periods [1,6]. However, these consequences are rarer in the third trimester [6].

## 2. Epidemiology

Parvovirus B19 is a common human pathogen, and more than 50% of the population will be infected during adulthood [7]. Although it occurs worldwide, the prevalence of parvovirus B19 infection varies by age and geography [8,9], with higher prevalence in developing countries and lower prevalence in isolated communities [10,11,12]. In terms of age, approximately 15% of preschool children, 50% of adults, and 85% of the elderly are seropositive for parvovirus B19 [13]. Pregnant women who do not have antibodies against the virus are as susceptible to parvovirus B19 infection as any other immunocompetent adult. Between 34% and 65% of pregnant women are not immune to parvovirus B19 at the beginning of pregnancy, and the incidence of seroconversion during pregnancy is estimated to be 1–15% in endemic periods and up to 13% in epidemic periods [14]. Furthermore, the highest rates of seroconversion in pregnant women, who were susceptible at the beginning of pregnancy, are found among teachers (16%) and housewives (9%) [15].

Parvovirus B19 can be transmitted by respiratory droplets, blood and blood product transfusions, bone marrow transplants, or transplacental passage, and the incubation period is approximately one week [16]. Viremia begins approximately 6 days after exposure and lasts up to one week. An infected person is infectious before the onset of symptoms and is unlikely to be infectious after the onset of rash, arthralgia, or arthritis. Serum samples are usually negative 7 days after the onset of illness, suggesting that patients with EI are probably in a period of reduced infectivity [17,18].

## 3. Maternal Infection—Clinical Presentation

Parvovirus B19 infection may be asymptomatic or may present with prodromal symptoms only. In some cases, the prodrome is followed by a subsequent phase with more specific symptoms, while in other cases, as in immunosuppressed or high-risk individuals, the infection may progress to a chronic form associated with long-term complications and sequelae [19,20]. However, parvovirus infection can be asymptomatic in more than 50% of non-pregnant women and almost 30–50% of pregnant women, respectively [21].

The incubation period varies from 4 to 14 days after exposure but can last up to 3 weeks. EI is the most classic sign of the disease and is more common in children. It is characterized by a “slapped face” appearance with a maculopapular rash on the face and/or trunk and extremities, which may present as a reticular rash [19,20].

The clinical course and immune response to parvovirus B19 is biphasic, with a second phase of symptoms presenting with rash, pruritus, or arthralgia approximately 17 to 18 days after vaccination [22,23]. Peripheral polyarthropathy of the hands, wrists, and knees is also characteristic and may be the only manifestation in pregnant women [24]. Polyarthropathy affects up to 50% of pregnant women and can last for several weeks to months [25]. More severe cases may result in aplastic crisis, this condition usually being self-limiting, and it is common in individuals with underlying chronic anemia such as sickle cell disease, HIV infection, and other inherited or acquired immunodeficiencies [26,27]. Parvovirus B19 IgG-positive individuals are generally considered immune to recurrent infection, but reinfection is possible [17].

Aplastic crisis is defined as a reticulocyte count below 50% of the baseline for at least two consecutive weeks. This event, although rare, is more common in patients who already have some form of chronic anemia (sickle cell, thalassemia, etc.). The main hypothesis for this outcome is that parvovirus particles are cytotoxic to erythrocyte precursor cells in the bone marrow. In addition, in patients with chronic anemia, the number of erythrocyte progenitor cells in the bone marrow is higher, which may facilitate the replication of B19 and explain the higher viremia observed in these patients during the acute phase of aplastic crisis. Aplastic crisis does not seem to be associated with gender or the presence of a rash [20].

## 4. Intrauterine Infection

### 4.1. Transmission

The rate of transmission of maternal parvovirus B19 infection to the fetus ranges from 17% to 33% [28], with an increased risk of transmission between 9 and 20 weeks of gestation, and most fetuses show spontaneous resolution of the infection without adverse perinatal outcomes [29,30]. Only acute parvovirus B19 infection may place the fetus at risk for anemia and hydrops [31].

### 4.2. Pathophysiology

Parvovirus B19 is not teratogenic, unlike viruses like rubella. However, fetal tissues—including hematopoietic cells in the liver, myocardium, endothelial cells, platelets, megakaryocytes, and fibroblasts—express the viral P antigen receptor, explaining the variety of fetal signs, especially anemia and hydrops [32].

The fetal liver is the primary hematopoietic organ from 9 to 24 weeks of gestation. The second trimester also sees the most rapid increase in the mass of fetal red blood cells, which increases more than 30-fold in number. However, the half-life of fetal red blood cells produced in this period is relatively short, approximately 45–70 days [33]. Therefore, the fetus is extremely vulnerable to any pause in red blood cell production during the second trimester and is more susceptible to the changes caused by parvovirus B19 [34]. This risk is greatly reduced in the third trimester when fetal hematopoiesis migrates to the bone marrow, and red blood cell lifespan increases.

### 4.3. Effects of Infection on Pregnancy

Pregnancy does not appear to affect the course of the parvovirus B19 infection, but the infection can affect the pregnancy and especially the fetus, as described below:

(1) Spontaneous abortion: The rate of spontaneous abortion decreases with gestational age at diagnosis, with a rate of 13% before 20 weeks’ gestation and 0.5% after 20 weeks’ gestation [28,30]. The reason for this difference remains unclear, but the largest study suggests that it may be related to multiple organ damage, which can occur in the absence of anemia or hydrops, the most classic findings associated with fetal infection [6].
(2) Non-immune hydrops: The most obvious manifestation of congenital parvovirus B19 infection is fetal hydrops. The risk of hydrops is directly related to the gestational age when maternal infection occurs. If the infection occurs in the first trimester, the risk of hydrops varies from less than 5% to about 10%. If the infection occurs between 13 and 20 weeks, the risk of hydrops drops to 5% or less. If the infection occurs after 20 weeks’ gestation, the risk of fetal hydrops is 1% or less [8,18,25].

**Figure 1:**
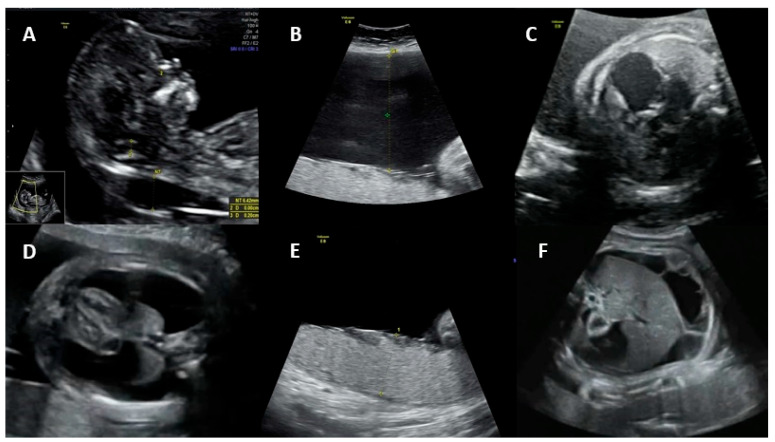
Main ultrasonographic findings of intrauterine parvovirus B19 infection: (**A**) increased nuchal translucency, (**B**) polyhydramnios, (**C**) cardiomegaly, (**D**) pleural effusion, (**E**) placentomegaly, and (**F**) ascites.

Fetal ultrasound signs associated with hydrops include ascites, skin edema, pleural and pericardial effusions, and placental edema. Possible mechanisms for the development of hydrops are (1) fetal anemia due to the virus crossing the placenta and infecting erythrocyte precursors in the fetal bone marrow, associated with a shorter fetal erythrocyte half-life (especially during the hepatic stage of hematopoiesis), contributing to severe anemia, hypoxia, and high-output heart failure; (2) fetal viral myocarditis resulting in heart failure and impaired liver function due to direct damage to hepatocytes and indirect damage from hemosiderin deposition [25,28,30]. Spontaneous improvement of fetal hydrops may occur in approximately 34% of cases by the time of delivery [35]. Figure 1 shows the main ultrasonographic findings of intrauterine parvovirus B19 infection.

Thrombocytopenia has been reported in up to 97% of transfused hydropic fetuses, with an incidence of severe thrombocytopenia (<50 × 10^9^ platelets/L) of up to 46% [6,35].

There is currently no evidence of an increased risk of human congenital anomalies in parvovirus B19 infection [29], although cases of central nervous system, craniofacial, musculoskeletal, and ocular malformations have been reported [36,37]. Table 1 shows the maternal and fetal clinical presentation of intrauterine parvovirus B19 infection.

## 5. Maternal Diagnosis

Systematic screening for parvovirus B19 infection is not recommended, and diagnostic testing is reserved for women with high suspicion of acute infection or known exposure [38,39]. Laboratory diagnosis of parvovirus B19 infection during pregnancy is mainly based on IgG and IgM antibody detection tests. The detection rate of infection remains around 80–90% when using radioimmunoassay (RIA) tests to capture antibodies or enzyme-linked immunosorbent assay (ELISA) [40].

IgM antibodies are detected early, by the end of the first week of infection [41], and can persist for approximately 140 days. IgG antibodies, as markers of past infections, can become positive a few days after IgM and remain positive for years [27]. Patients with IgG-positive, IgM-negative serologic test results indicate previous viral exposure and possibly immunity, suggesting that they will unlikely contract the disease in future. [27,39,42]. Caution should be exercised in interpreting the absence of positive IgM from 8 to 12 weeks after acute infection because of the possibility of rapid IgM clearance and false-negative results.

In cases where both parvovirus B19 IgM and IgG serologies are negative, it is assumed that the pregnant woman has not been exposed to the virus and is therefore susceptible to infection. In non-immune pregnant women recently exposed to the virus, the viral incubation period should be considered, and serology should be repeated two to four months after exposure, also considering polymerase chain reaction (PCR) [38,39,42,43].

Both positive IgG and IgM serologies may represent a recent infection or a late infection of up to 6 months. To differentiate between the two hypotheses, it is recommended to repeat the serology and compare the IgG titers, since they will be increasing in case of a recent infection [39,43]. Another possibility is IgM positivity only, suggesting a very recent infection, or even a false-positive result. IgG positivity on repeated serology one to two weeks after the initial test confirms a recent infection [39,42].

PCR assays for parvovirus B19 have high specificity for acute primary infection but have a short window of positivity, which limits their use in practice. Viremia occurs between 5 and 10 days after exposure and usually persists for about 7 days, a period that often precedes the onset of symptoms and serologic positivity [39]. In asymptomatic patients, because the day of infection is unknown, this test cannot be used as a gold standard [41]. The use of these tests may be useful in certain situations, for example to achieve maximum diagnostic sensitivity in patients with a history of recent exposure to parvovirus B19 and negative initial serologies [39]. Although not necessary to detect acute maternal infection, other techniques such as electron microscopy, viral DNA detection, and nucleic acid probe hybridization assays are available. It is not possible to culture the virus in regular culture media, and therefore, culture for parvovirus B19 is not used as a diagnostic test [42]. Table 2 shows the maternal classification of intrauterine parvovirus B19 infection according to laboratory tests.

## 6. Fetal Diagnosis

### 6.1. Diagnosis of Infection

Fetal hydrops associated with or without moderate/severe anemia in absence of a relevant cause should prompt amniocentesis followed by PCR to detect parvovirus B19 [44]. In these cases, maternal serology is useful only if both IgG and IgM are negative, ruling out maternal and therefore fetal infection. In all other cases, maternal serology is compatible with possible fetal infection, including positive IgG and negative IgM [39].

All diagnostic methods have limitations. The use of PCR is only useful in the viremic period, when viral particles can be traced [45]. On the other hand, the immune response of fetal IgG and IgM is unpredictable due to the immaturity of the fetal immune system, so fetal serology is rarely used and less useful for diagnosis [39,43]. PCR for parvovirus B19 can be performed on fetal blood samples obtained by cordocentesis, but this technique has a fetal loss rate of approximately 1%. Amniotic fluid sampling has a higher detection rate with less chance of complications and is therefore preferred for diagnosis [39,42]. In postmortem samples, electron microscopy can detect intranuclear inclusions or viral particles, orienting the diagnosis [45].

### 6.2. Diagnosis of Fetal Anemia

In pregnant women who have recently been infected with parvovirus B19, and the infection has been demonstrated, the risk of maternal–fetal transmission is high, and a close US follow-up with a specialist obstetrician must be planned. The fetus must be closely monitored to detect fetal anemia early, which may arise from 8 to 12 weeks after infection, and then every one to two weeks [39,42].

Fetal anemia is initially monitored non-invasively by assessing peak systolic velocity of the middle cerebral artery (PSV-MCA) using Doppler ultrasound. Moderate/severe fetal anemia is highly indicative of the presence of a PSV-MCA greater than 1.5 times the median (MoM) at 18 weeks of gestation or later [46,47,48,49].

**Figure 2:**
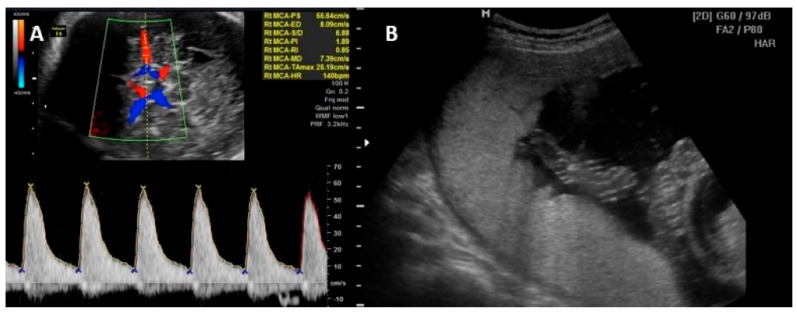
(**A**) Peak of systolic velocity of the middle cerebral artery Doppler measurement technique. (**B**) Intrauterine transfusion with the needle in the vein at level of umbilical cord insertion on placenta.

Parvovirus B19 infection is unlikely the cause of fetal anemia in the absence of ultrasound changes suggestive of fetal sequelae 8 to 12 weeks after possible exposure. Cordocentesis with fetal blood sampling to determine fetal hematocrit and intrauterine transfusion may be required in cases of fetal hydrops or severe fetal anemia [46,47,48,49]. Other signs of fetal parvovirus B19 infection include fetal ascites and cardiomegaly. Generalized edema and pericardial effusion occur in more advanced stages of the disease. Hyperechogenic bowel, meconium peritonitis, increased nuchal translucency in the first trimester, and amniotic fluid abnormalities have also been reported as US anomalies [50,51,52]. Figure 2 illustrates the PVS-MCA measurement and the intrauterine transfusion.

## 7. Prognosis

Although parvovirus B19 infection contributes little to the overall increase in the rate of fetal demise, infection has been found to increase the risk of fetal death, spontaneous abortion, and stillbirth compared with uninfected women [53,54]. The most important determinant of mortality and adverse perinatal outcomes is the presence of fetal hydrops, with a mortality rate of approximately 29%. Spontaneous resolution of anemia occurs in approximately 5.2% of fetuses with hydrops compared to approximately 49.6% of non-hydropic fetuses [34].

Mortality in parvovirus B19 infection can be dependent on the gestational age. Pregnant women diagnosed with the infection in the first trimester had an abortion rate of about 13% compared to 9% in patients diagnosed between 13 and 20 weeks of gestation. The majority of fetal deaths (80%) occurred up to 4 weeks after infection [55].

Parvovirus B19 infection itself, in the absence of hydrops or significant fetal anemia, does not appear to cause long-term neurologic morbidity, but further studies are needed [6,26]. However, severe anemia or hydrops may be an independent long-term risk factor for neurologic sequelae. In reported cases of survivors of fetal hydrops, neurological complications include ventriculomegaly, polymicrogyria, cerebral heterotopia, and other anomalies of the central nervous system [56,57]. The prevalence of anomalies in brain imaging scans is around 9.8%. The risk of neurodevelopmental abnormalities is almost 10% in children with a history of fetal hydrops due to parvovirus B19 infection [34]. Neurodevelopmental disorders, although rare, can also occur after intrauterine transfusion in up to 12% of children, and these disorders can range from mild cognitive delay, mild fine motor impairment to severe developmental delay with neurological abnormalities such as ataxia, hypertonia, and cerebral palsy [58]. Table 3 presents the perinatal outcomes of intrauterine parvovirus B19 infection.

## 8. Primary Prevention

Given that there is no specific treatment for parvovirus B19 infection, prophylaxis plays a key role in minimizing the morbidity of the disease. Preventing maternal exposure is the main focus of this prophylaxis, and it is debatable whether pregnant women susceptible to infection should avoid the high-risk population [46].

In endemic areas, pregnant women with school-age family members and those who work with school-age children should be informed about transmission of parvovirus B19 and adopt precautional measures to prevent its spreading. Hygiene measures, such as hand washing, are suggested to minimize the risk of infection, as diffusion can occur by droplets [42].

## 9. Secondary Prevention

To date, there is no vaccine for parvovirus B19 released for use in humans, although vaccine trials are being carried out [59,60,61,62]. An in vivo study was discontinued after the second dose of vaccination due to adverse skin reactions in some patients. Most patients developed neutralizing antibodies to parvovirus B19 after two doses of the vaccine, suggesting some degree of immunization [62]. Further studies and trials are needed, also taking into account the recent advances in medical technology.

## 10. Treatment

Treatment of a fetus infected with parvovirus B19 mainly consists of management of fetal anemia. Cases of mild-to-moderate anemia are generally well tolerated and resolve without sequelae and do not require invasive interventions during the fetal period. Severe anemia, although uncommon, may result in fetal hydrops and death.

The tool used to determine the degree of anemia noninvasively, without the need for fetal blood sampling, is the PVS-MCA, which when elevated (>1.5 MoM) is highly suggestive of moderate/severe anemia. Other ultrasound parameters such as fetal skin edema, ascites, or pleural or pericardial effusions may occur and are suggestive of severe anemia, but these are also parameters found later in the fetus [39].

If US findings are suggestive of severe fetal anemia, fetal hematocrit should be determined by cordocentesis. However, severe thrombocytopenia can also be present, which could lead to exsanguination at the time of intrauterine red blood cell transfusion. For this reason, the platelet count should be determined, and platelets should be available for intrauterine transfusion at the time of any fetal procedure [39]. If fetal status is confirmed, intrauterine fetal blood transfusion is indicated [42].

If pregnancy is close to term, delivery should be considered as the first option, and the use of corticosteroids to accelerate lung maturation should be administered in case of necessity, as not contraindicated [63].

### 10.1. Intrauterine Transfusion

Intrauterine red blood cell transfusion is indicated to prevent fetal death due to severe anemia. Ideally, this procedure should be carried out on fetuses between 18 and 35 weeks of gestation, because transfusions carried out before 18 weeks have important technical limitations, and transfusions carried out after 35 weeks have greater fetal risks than the delivery. Fetal survival rates with intrauterine transfusion remain at around 82%, compared to survival rates of 55% in fetuses not transfused [64]. Two or three intrauterine transfusions may be necessary to resolve fetal anemia and hydrops, which usually occurs 3 to 6 weeks after the first treatment [65].

Due to fetal myocarditis, the degree of hydrops may not correlate with fetal hemoglobin, and fetal echocardiography is useful in these cases [42]. PSV-MCA should be used as monitoring after intrauterine transfusion for anemia and fetal complications, even though its prediction for anemia decreases [39].

### 10.2. Intravenous Immunoglobulin

Intravenous immunoglobulin has been used to treat acute parvovirus B19 infection in immunocompromised patients. The use of intravenous immunoglobulin in pregnancy has been reported in a few case reports and only in a few in vitro studies [66,67,68]. One case reported fetal intraperitoneal immunoglobulin injection with a good outcome, in which the fetal ascites and pericardial effusion resolved after approximately 5 days with a complete resolution at 22 weeks of gestation. At birth, the newborn was in apparent good health and no parvovirus B19 DNA was isolated in the blood sample [67].

Due to the lack of relevant studies, immunoglobulin is not recommended as fetal therapy, but it may be considered as a promising alternative.

### 10.3. Delivery Room and Postnatal Management of the Hydropic Child

Delivery and management of a pregnant women with a history of acute parvovirus B19 infection should be performed in a tertiary center with an experienced multidisciplinary team and specialized neonatal care. The majority of hydropic neonates require respiratory support and mechanical ventilation. Several factors may contribute to respiratory failure, including pulmonary hypoplasia, pulmonary edema, pleural effusion, and ascites.

**Figure 3:**
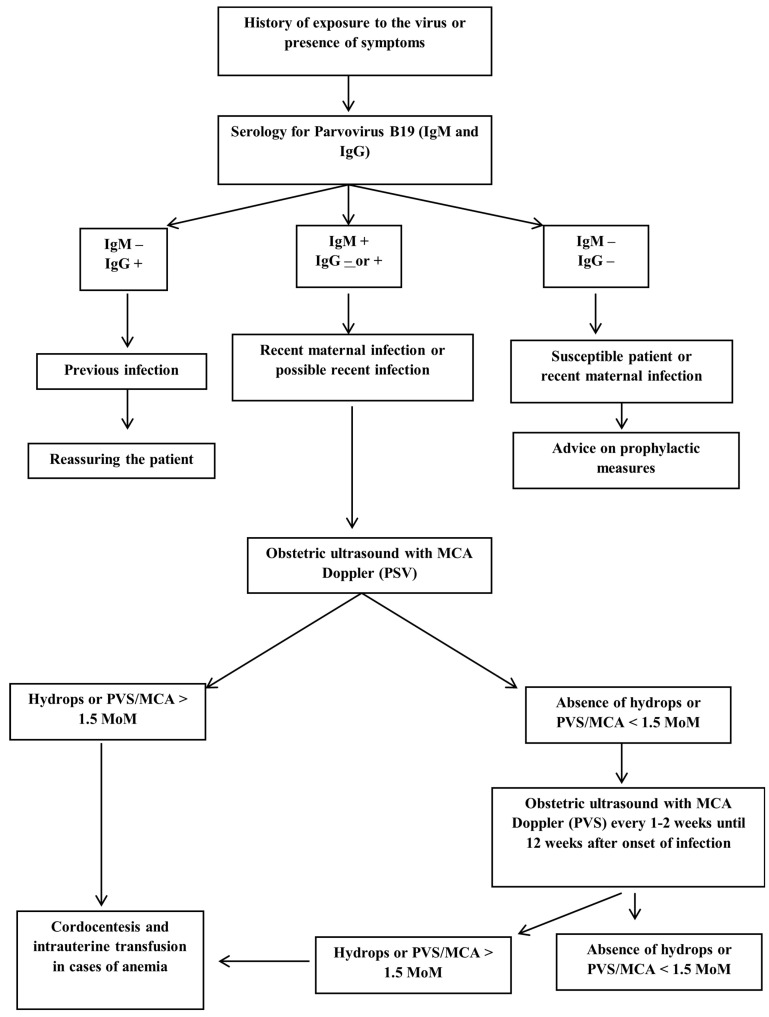
Flowchart showing the path followed from the maternal serology to the fetal treatment of parvovirus B19 infection.

Some maneuvers may be necessary to facilitate cardiopulmonary resuscitation, such as abdominal paracentesis and thoracentesis of pleural effusions. Isovolumetric transfusion in neonates with severe anemia and cardiovascular instability is a possible treatment [6]. Figure 3 shows the flowchart from the maternal serology to the fetal treatment of parvovirus B19 infection.

## 11. Conclusions

Parvovirus B19 is a virus transmitted via the respiratory tract, blood products, or the transplacental route. As this last transmission route exists, it can result in infection and cause fetal consequences. Most immunocompetent patients (pregnant or non-pregnant) have mild symptoms associated with the infection, mainly respiratory symptoms and maculopapular rash (erythema infectiosum). However, when there is fetal infection, there can be fetal anemia and even, in more severe cases, fetal hydrops. Fortunately, these repercussions are rare, and most pregnancies with parvovirus B19 infection are uneventful. The symptoms of anemia and hydrops occur due to the tropism of the virus for the blood precursor cells in the fetus, which is more vulnerable because it has a more susceptible hematopoietic system.

There is no recommendation for universal screening of pregnant women during prenatal care for parvovirus B19 infection, but fetal identification for the correct management of affected fetuses has been shown to be a strategy for improving perinatal outcomes. As most pregnancies infected with parvovirus B19 have a favorable outcome, indications for invasive prenatal diagnosis are rare, and only indicated if there are definite signs of fetal anemia or hydrops. Fetal infection can be diagnosed using PCR in amniotic fluid, but this method is limited because it may only be positive during the viremic period of the disease.

Fetal management of parvovirus B19 infection consists mainly of checking for signs of hydrops (ascites, skin edema, pleural and pericardial effusions, placental edema, etc.) and also for moderate/severe fetal anemia using the PVS-MCA. If the PVS-MCA is >1.5 MoM and the gestational age is between 18 and 35 weeks, intrauterine transfusion by cordocentesis is indicated. Fetal intrauterine transfusion for hydropic fetuses improves survival rates. More than one transfusion may be necessary, and anemia and/or hydrops usually resolve between 3 and 6 weeks after the first intrauterine transfusion.

## Figures and Tables

**Table 1:** Clinical maternal and fetal presentation of the intrauterine parvovirus B19 infection.

Binomial	Clinical Presentation
Maternal	
	Asymptomatic
	Erythema infectiosum
	Arthropathy
	Myocarditis
	Anemia
	Leukopenia
	Thrombocytopenia
Fetal	
	Anemia
	Thrombocytopenia
	Myocarditis
	Pleural effusion
	Pericardial effusion
	Ascites
	Non-immune hydrops Neurological impairment Congenital anomalies
	Intrauterine fetal death

**Table 2:** Classification of maternal intrauterine parvovirus B19 infection according to laboratory tests.

Classification	Definition
**Recent infection**	IgG and IgM negative previously with results showing seroconversion during pregnancy OR IgG and IgM positive, repeat test in 2 weeks with increasing IgG titers OR IgG negative with IgM positive, repeat test in 2 weeks and IgG becomes positive
**Previous infection**	IgG positive with IgM negative OR IgG and IgM positive, repeat test in 2 weeks with plateau IgG titers
**Non-immune (susceptive)**	IgG e IgM negative
**False positive**	IgG negative with IgM positive, test repeated in 2 weeks and IgG remains negative
**Congenital infection**	PCR positive for parvovirus B19 in fetal blood collected by cordocentesis

**Table 3:** Perinatal outcomes of intrauterine parvovirus B19 infection.

Perinatal Outcome	Incidence
Asymptomatic infection	50%
Vertical transmission	30%
Abortion and intrauterine fetal death	
<13 weeks	13%
13–20 weeks	9%
>20 weeks	1%
Non-immune fetal hydrops	
<13 weeks	5–10%
13–20 weeks	5%
>20 weeks	<1%
Spontaneous anemia resolution	
Hydrops fetalis	5.2%
Non-hydrops fetalis	49.6%

## Data Availability

Not applicable.
